# Comparing variants related to chronic diseases from genome-wide association study (GWAS) and the cancer genome atlas (TCGA)

**DOI:** 10.1186/s12920-023-01758-7

**Published:** 2023-12-19

**Authors:** Soohyun Jeon, Chaewon Park, Jineui Kim, Jung Hoon Lee, Sung-yune Joe, Young Kyung Ko, Jeong-An Gim

**Affiliations:** 1 Department of Brain and Cognitive Engineering, Korea University, Seoul, 02841 South Korea; 2 School of Biomedical Engineering, Korea University, Seoul, 02841 South Korea; 3 Interdisciplinary Program in Precision Public Health, Korea University, Seoul, 02841 South Korea; 4 Department of Microbiology, Institute for Viral Diseases, College of Medicine, Korea University, Seoul, 02841 South Korea; 5 Department of Pharmacology, College of Medicine, Korea University, Seoul, 02841 South Korea; 6 Division of Pulmonary, Allergy and Critical Care Medicine, Department of Internal Medicine, Korea University Guro Hospital, Seoul, 08308 South Korea; 7 Department of Medical Science, Soonchunhyang University, Asan, 31538 South Korea

**Keywords:** Cancer, Chronic diseases, Variants, Genome-wide association study, 1000 genomes project

## Abstract

**Background:**

Several genome-wide association studies (GWAS) have been performed to identify variants related to chronic diseases. Somatic variants in cancer tissues are associated with cancer development and prognosis. Expression quantitative trait loci (eQTL) and methylation QTL (mQTL) analyses were performed on chronic disease-related variants in TCGA dataset.

**Methods:**

MuTect2 calling variants for 33 cancers from TCGA and 296 GWAS variants provided by LocusZoom were used. At least one mutation was found in TCGA 22 cancers and LocusZoom 23 studies. Differentially expressed genes (DEGs) and differentially methylated regions (DMRs) from the three cancers (TCGA-COAD, TCGA-STAD, and TCGA-UCEC). Variants were mapped to the world map using population locations of the 1000 Genomes Project (1GP) populations. Decision tree analysis was performed on the discovered features and survival analysis was performed according to the cluster.

**Results:**

Based on the DEGs and DMRs with clinical data, the decision tree model classified seven and three nodes in TCGA-COAD and TCGA-STAD, respectively. A total of 11 variants were commonly detected from TCGA and LocusZoom, and eight variants were selected from the 1GP variants, and the distribution patterns were visualized on the world map.

**Conclusions:**

Variants related to tumors and chronic diseases were selected, and their geological regional 1GP-based proportions are presented. The variant distribution patterns could provide clues for regional clinical trial designs and personalized medicine.

## Introduction

Chronic diseases are defined as conditions that last 1 year or more and require medical intervention, restrict activities of daily living, or both. Chronic diseases include hypertension, diabetes, hyperlipidemia, and many associations with cancer have also been known [1–3]. Genome-wide association studies (GWASes) have been used as a research approach to understand chronic diseases. GWAS can help to understand the risk of chronic diseases and specific characteristics, such as cancer morbidity in an individual [4, 5]. Until now, GWAS results have been open to the public, and optimal secondary applications have been presented.

Variants indicate alterations in DNA nucleotide sequences. There are single-base pair substitutions, insertions or deletions (INDEL), and structural variations. The somatic variant refers to every variant in cells, except germ cells. Unlike germline variants, somatic variants are not inherited, and reflect genomic instability [6, 7]. Next-generation sequencing (NGS) is widely used to obtain nucleotide sequence data from cancer cells. Variants of cancer cells enable targeted therapy according to genotype. An expression quantitative trait locus (eQTL) is a variant that explains differences in gene expression patterns. A methylation QTL (mQTL) is also a variant related to the different beta values of CpG sites in the genome. eQTL and mQTL are variants of the GWAS results and are independent variables for gene expression and DNA methylation level as dependent variables [8, 9]. Many eQTL and mQTL signals have been found in chronic disease samples, and biomarkers for prognosis in cancer patients are needed for variants related to chronic diseases.

The Cancer Genome Atlas (TCGA) is a project that started in 2005 to integrate and accumulate cancer genetic variants, gene expression, and DNA methylation data using bioinformatics technologies [10]. TCGA database was provided by the National Cancer Institute of the United States. TCGA Data Portal provides researchers with a platform to search, download, and analyze cancer genomic data. TCGA provides clinical data (subtype, survival, and recurrence) and three types of omics data (variant, expression, and methylation) for 7648 patients and 33 types of cancers. Therefore, by properly processing clinical and omics datasets for the purpose of analysis, it is possible to accurately identify the factors that explain the traits of cancer [11–14].

The 1000 Genomes Project (1GP) was launched to assess human genetic variation by ethnic groups. The pilot phase and the “phase 3” were completed as 1092 and 2504 genomes, respectively. In 1GP Phase 3, 26 populations were collected [15]. The 1GP helps explain the genetic variants that occur at a population frequency of 1% or more. It also contributes to the development of preventive medicine using genetic variants found in a specific ethnic group [16, 17]. The genomic composition of the population distributed by region was changed by the evolutionary process because selective pressure and SNP density differed by ethnic group. Clinical approaches, such as disease susceptibility and drug response prediction, are also available in this region [18].

In this study, eQTL and mQTL studies were combined with GWAS to identify genes associated with cancer prognosis, and variants related to cancer were found in TCGA. Relevance to the 1GP for merged variants was confirmed. The relationship between cancer and chronic diseases was confirmed, and regional differences were visualized using 1GP data.

## Methods

### Data acquisition from TCGA and LocusZoom

**Fig. 1:**
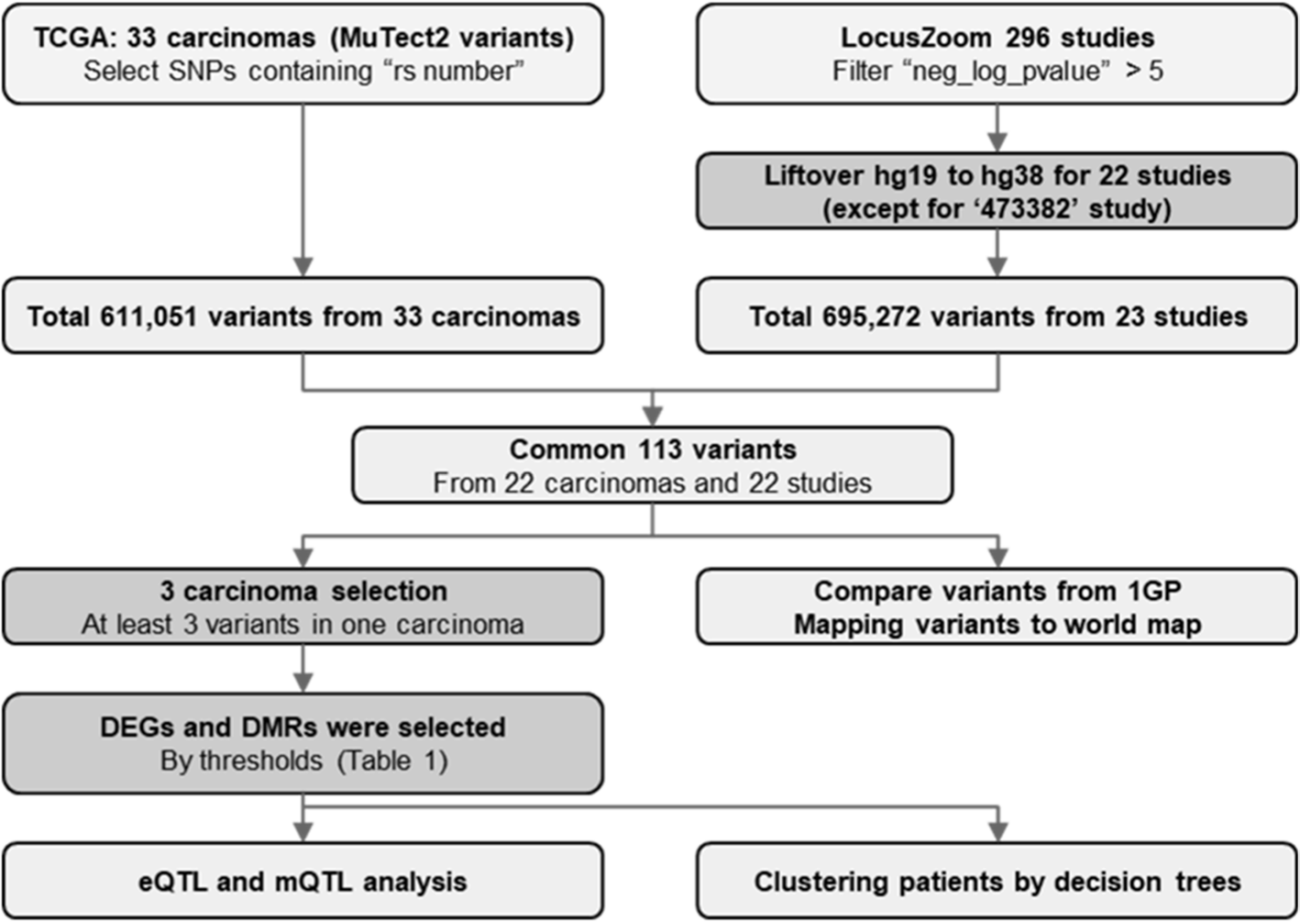
Process of this study. Description of the input and output data is shown as light gray, and the data process is shown as dark gray

The 33 omics and clinical data of this study were downloaded from TCGA dataset. Downloads and data processing were performed using the “GDCquery” function of the R package “TCGAbiolinks” [19]. All analyses were performed using R package version 4.1.1. GWAS datasets were downloaded from LocusZoom (https://my.locuszoom.org/) [20] and each study name was identified as the URL number. This study was approved by the Institutional Review Board (IRB) of Korea University (approval number: KUIRB-2020-0191-01) and was performed in accordance with the Declaration of Helsinki. All processes of this study are presented as a flowchart (Fig. 1).

### DEG and DMR selection

TCGA RNA-seq data revealed the expression levels of 56,457 genes. Analysis with the Illumina 450 k chip in TCGA identified approximately 450,000 CpG sites. Differentially expressed genes (DEGs) and differentially methylated regions (DMRs) were selected between the patients with and without variants. The fold change and *p*-value of the selected DEGs and DMRs are presented as volcano plots, and the expression level and DNA methylation level of genes above a certain threshold are presented as heatmaps. Expression and DNA methylation levels are presented as boxplots for each genotype.

### Visualization of variant data

A variant heatmap was presented using the “Heatmap” function of the “ComplexHeatmap” package, and a waterfall plot for variants was presented using the “oncoPrint” function [21]. The heatmap for DEG and DMR utilized the “pheatmap” package. In the case of the volcano plot, an in-house source was coded using “plot,” the default function of R.

### Validation at the 1000 genomes projects for variants

The 1000 Genomes Project (Phase 3) data were downloaded from Google Cloud Life Sciences (https://cloud.google.com/life-sciences/docs/resources/public-datasets/1000-genomes). The total data consisted of 84,801,856 variants of 69,006 dbSNP rs numbers for 2504 individuals [15]. The 1000 Genomes Project variants matching the dbSNP rs number of TCGA variants were selected using the “filter” function of the “dplyr” R package.

A world map was presented using the “map” function of the “maps” R library. The “floating.pie” function of the “plotrix” R library was used to present the location and variant proportion of each population. The global positioning system (GPS) information for each population was obtained from GitHub (https://github.com/sinarueeger/map-1000genomes).

### Machine learning approaches of clinical data, DEG, and DMR results

Integrative analysis was performed for the selected DEGs and DMRs using the clinical data. Decision tree is the machine learning approach that used for both classification and regression tasks. The decision tree algorithm recursively divides the dataset into subsets based on the values of different attributes. The aim is to create that are as pure as possible with respect to the target variable. Model design and visualization for decision trees were performed using “rpart” and “rpart.plot” libraries. The models were fitted and tuned for each cancer. The decision tree model was presented by selecting the cost complexity pruning (cp) value with minimum error.

## Results

### TCGA variants processing

**Fig. 2:**
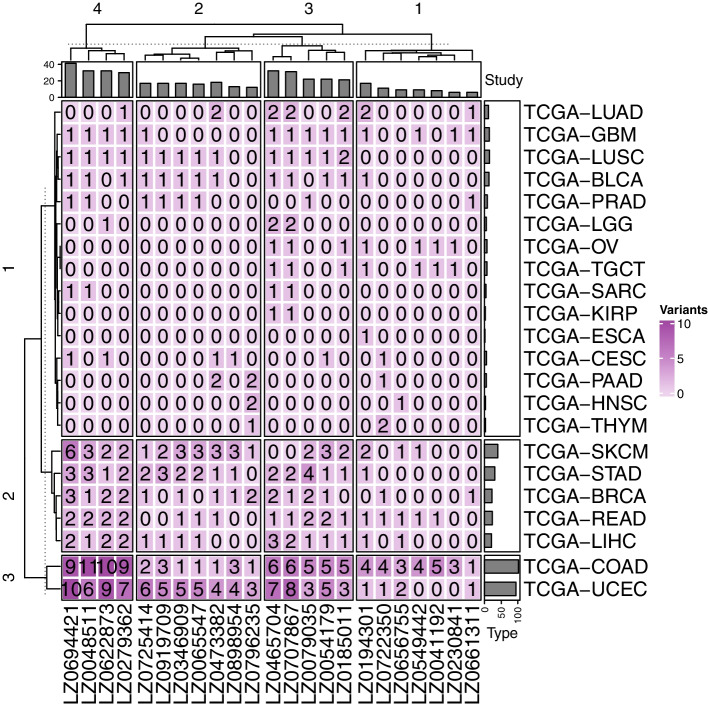
Patterns of common variants between TCGA 22 cancer datasets and LocusZoom 23 datasets. TCGA 22 cancer were divided into three row clusters, and LocusZoom 23 datasets were grouped to four column clusters. Each four-column cluster number was indicated at the top of the columns. Three-row cluster numbers were indicated at the left of the rows

In 22 cancers out of a total of 33 cancers, at least one variant overlapped with the variants found in the 23 datasets obtained from LocusZoom. Over 20 variants overlapped in seven cancers (Fig. 2), and at least one variant was observed in 10 or more patients in four cancer types (TCGA-COAD, TCGA-UCEC, TCGA-SKCM, and TCGA-STAD). In TCGA-SKCM samples, only two of the 103 patients had variants. We excluded TCGA-SKCM from the DEG and DMR analyses because *t*-test was performed using at least three samples per group in DEG and DMR analysis (Table 1).

**Table 1:** Descriptions of TCGA dataset

	TCGA-COAD	TCGA-STAD	TCGA-UCEC	TCGA-SKCM
RNA-seq				
Total genes	56,457	56,457	56,457	56,457
RNA-seq, no variant group	429	364	504	101
RNA-seq, variant group	21	9	33	2
Total RNA-seq samples	450	373	537	103
Threshold	PV < 0.01 & |FC| > 0.2	PV < 10^−10^ & |FC| > 0.3	PV < 0.01 & |FC| > 0.2	(No analysis)
Methylation 450 k				
Total CpG sites	118,342	373,352	379,215	380,110
Met450, no variant group	269	384	398	103
Met450, variant group	20	9	27	2
Total Met450 samples	289	393	425	103
Threshold	PV < 10^−12^ & |FC| > 0.2	PV < 10^−12^ & |FC| > 0.2	PV < 10^−12^ & |FC| > 0.2	(No analysis)

### Common variant selection of TCGA and LocusZoom

The TCGA single nucleotide variation (SNV) dataset from 33 cancers and variants satisfying log10 (p-value) > 5 were selected from 230 GWAS datasets. The two datasets were merged as “merge” R default function by “rs number.” For the commonly detected “rs number,” the number of patients with variants for each of 33 cancers was counted. TCGA 22 cancers found in at least one of the LocusZoom variants were presented as a heatmap (Fig. 2).

**Fig. 3:**
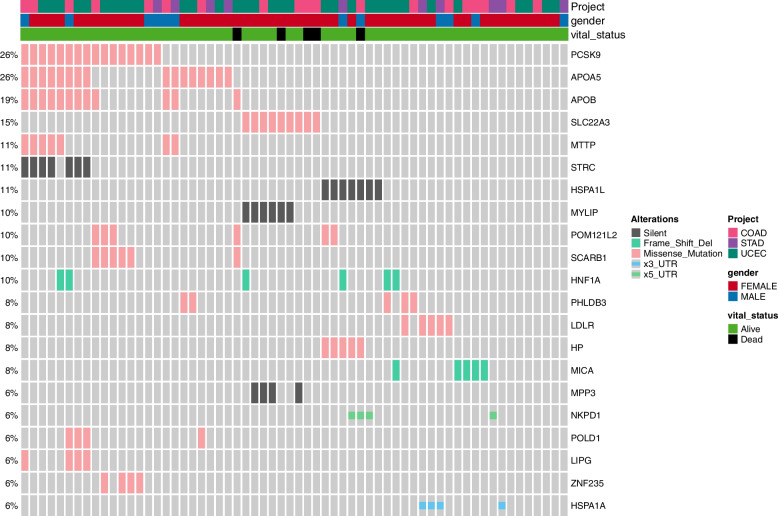
Waterfall plot of 21 variants matched to LocusZoom 13 studies from TCGA 62 samples from three cancer (COAD, STAD, and UCEC). Three column annotation bars indicate cancer type, gender, and survival status

Sixty variants found in three cancers of TCGA and 13 studies of LocusZoom were selected. A waterfall plot was presented for 21 mutations, with at least 4 mutations found in 62 patients from TCGA (Fig. 3). Eleven variants were commonly found in at least six mutations in TCGA and LocusZoom (Table 2).

**Table 2:** Most of the eleven variants presented were found six or more times in the TGCA or LocusZoom. In the presented LocusZoom studies, at least one variant satisfying -log10(*p*-value) > 5 was discovered

dbSNP rs number	Gene symbol	cancer	GWAS studies	Location (hg19)	Ref	Alt	Classification	-log10(PV) range
rs1060901	MYLIP	COAD (*n* = 1)	LZ0048511, LZ0054179, LZ0079035, LZ0279362, LZ0622873, LZ0694421 (*n* = 6)	chr6:16145242	C	T	Silent	28.02488-36.0667 (*n* = 6)
rs113337987	MTTP	COAD (*n* = 1)	LZ0048511, LZ0054179, LZ0079035, LZ0279362, LZ0622873, LZ0656755, LZ0694421 (*n* = 7)	chr4:99611445	G	A	Missense_Mutation	8.078993-13.79644 (*n* = 7)
rs12438025	STRC	COAD (*n* = 1)	LZ0041192, LZ0185011, LZ0194301, LZ0230841, LZ0465704, LZ0549442, LZ0707867 (*n* = 7)	chr15:43600649	G	C	Silent	13.99787-30.17285 (*n* = 7)
rs141502002	PCSK9	STAD (*n* = 1), UCEC (*n* = 1)	LZ0048511, LZ0065547, LZ0079035, LZ0346909, LZ0694421, LZ0725414, LZ0898954, LZ0919709 (*n* = 8)	chr1:55058549	C	T	Missense_Mutation	7.713815-19.52549 (*n* = 8)
rs151135411	SLC22A3	UCEC (*n* = 1)	LZ0048511, LZ0054179, LZ0065547, LZ0079035, LZ0279362, LZ0346909, LZ0622873, LZ0656755, LZ0694421 (*n* = 9)	chr6:160410764	G	A	Missense_Mutation	6.27433-31.41669 (*n* = 9)
rs2075799	HSPA1L	COAD (*n* = 1)	LZ0048511, LZ0185011, LZ0194301, LZ0230841, LZ0549442, LZ0725414, LZ0919709 (*n* = 7)	chr6:31810752	C	T	Silent	7.458832-19.77592 (*n* = 7)
rs3135506	APOA5	COAD (*n* = 1)	LZ0041192, LZ0048511, LZ0054179, LZ0079035, LZ0185011, LZ0194301, LZ0230841, LZ0279362, LZ0465704, LZ0549442, LZ0622873, LZ0656755, LZ0694421, LZ0707867, LZ0722350, LZ0898954 (*n* = 16)	chr11:116791691	G	C	Missense_Mutation	6.043769-Inf (*n* = 16)
rs41269255	POM121L2	COAD (*n* = 1)	LZ0048511, LZ0279362, LZ0622873, LZ0656755, LZ0694421, LZ0796235 (*n* = 6)	chr6:27309272	C	T	Missense_Mutation	5.999566-16 (*n* = 6)
rs41288783	APOB	STAD (*n* = 1)	LZ0048511, LZ0054179, LZ0065547, LZ0079035, LZ0279362, LZ0346909, LZ0622873, LZ0694421, LZ0725414, LZ0919709 (*n* = 10)	chr2:21019741	G	A	Missense_Mutation	20.4329-76.95933 (*n* = 10)
rs74830677	SCARB1	UCEC (*n* = 1)	LZ0048511, LZ0279362, LZ0465704, LZ0707867, LZ0725414, LZ0919709 (*n* = 6)	chr12:124800125	G	A	Missense_Mutation	5.07808-20.47277 (*n* = 6)
rs762703502	HNF1A	COAD (*n* = 4), UCEC (*n* = 2)	LZ0656755 (*n* = 1)	chr12:120994312	CG	C	Frame_Shift_Del	7.290137 (*n* = 1)

Locuszoom ID means the url of each study; e.g. LZ0048511 is linked to https://my.locuszoom.org/gwas/48511/

**Fig. 4:**
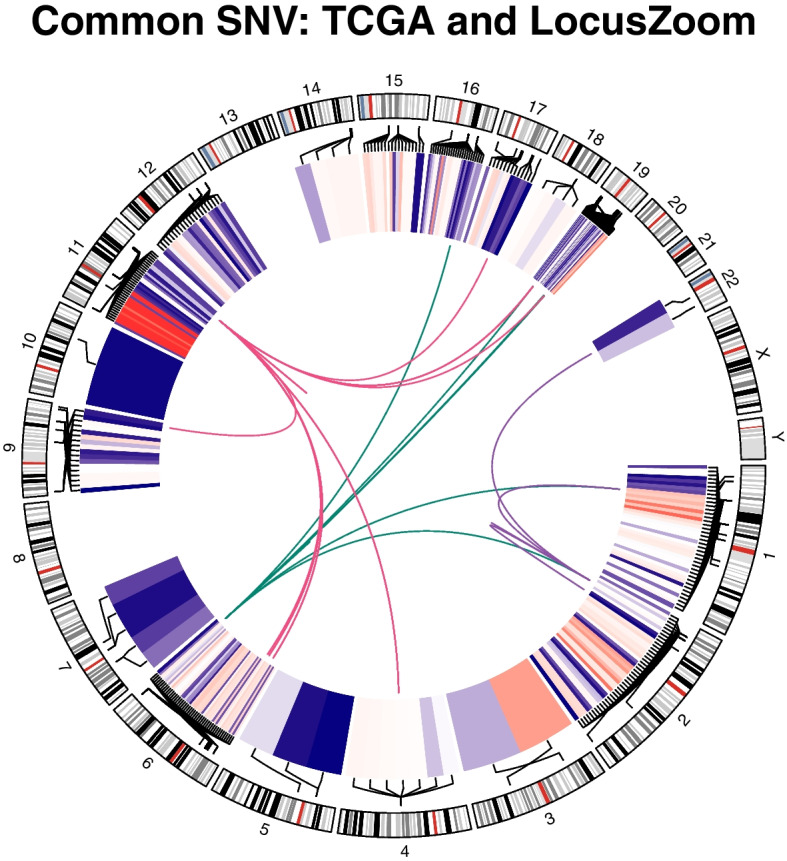
Circos plot of common variants between TCGA cancer and GWAS data. Linked lines of the same color mean the same cancer

The chromosomal locations of common variants between TCGA and LocusZoom data are presented in a Circos plot (Fig. 4). Among them, we linked the variants of UCEC, COAD, and STAD cancers of interest. UCEC was most common on chromosome 6, COAD on chromosome 11, and STAD on chromosome 2. The connection showed a relationship between the other variants and the most abundant variant of each cancer species.

### Variant distributions from 1000 genomes project data

**Fig. 5:**
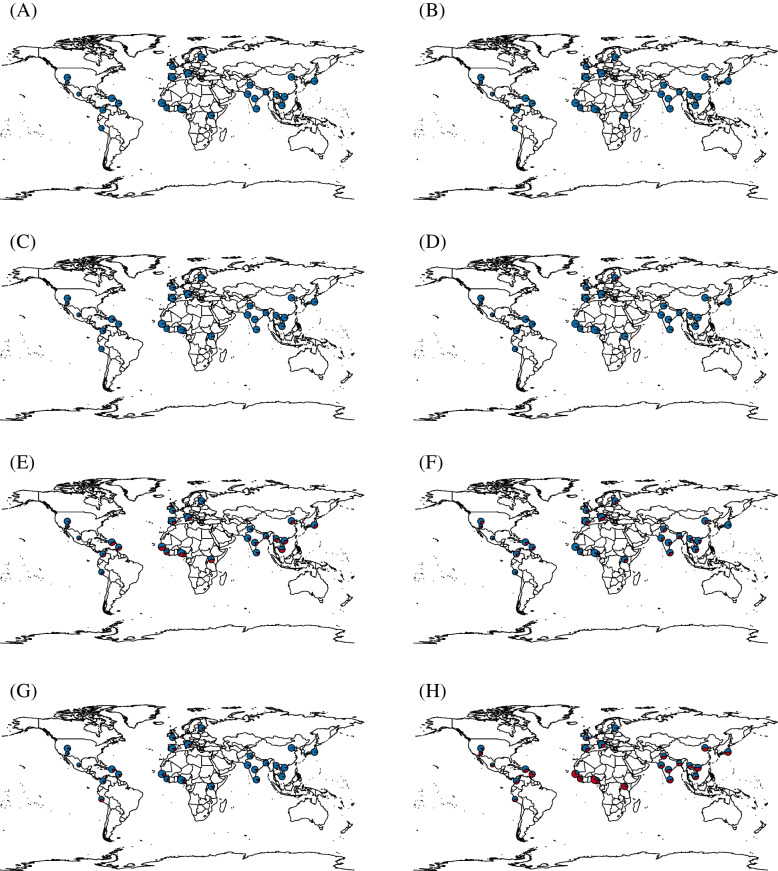
Geological locations and eight variant proportions of 26 populations from the 1000 Genomes Project (phase 3). **A** Detected in TCGA-STAD and -UCEC, rs141502002 in PCSK9 gene. **B** Detected in TCGA-STAD, rs41288783 in APOB gene. **C** Detected in TCGA-COAD, rs113337987 in MTTP gene. **D** Detected in TCGA-COAD, rs1060901 in MYLIP gene. **E** Detected in TCGA-COAD, rs2075799 in HSPA1L gene. **F** Detected in TCGA-COAD, rs41269255 in POM121L2 gene. **G** Detected in TCGA-COAD, rs3135506 in APOA5 gene. **H** Detected in TCGA-COAD, rs12438025 in STRC gene

Eight of the 11 variants were identified from the results of the 1000 Genomes Project (Phase 3). A total of 26 population variant proportions were identified and are displayed on a global map (Fig. 5). In the case of rs141502002, located in the PCSK9 gene, it was discovered in patients with STAD and UCEC, and was discovered in eight studies of LocusZoom. Nevertheless, low variant proportions were observed overall (Fig. 5a). The rs41288783 variant located in the APOB gene was also included in two studies by LocusZoom, including patients with STAD, but showed a low variant proportion overall (Fig. 5b). The rs113337987 variant located in the MTTP gene was found in COAD patients and LocusZoom 7 studies and showed slightly more variant proportions in the Caribbean, South America, and Southern Europe (Fig. 5c). The rs1060901 variant located in the MYLIP gene was found in COAD and LocusZoom 6 studies and was found in Europe (Fig. 5d). The rs2075799 variant located in the HSPA1L gene was found in Africa and Southeast Asia, and was found in COAD and seven LocusZoom studies (Fig. 5e). rs41269255, found in Europe, is located in the POM121L2 gene and was found in COAD in six studies (Fig. 5f). rs3135506 of the APOA5 gene, found in 16 studies of COAD and LocusZoom, showed low proportions, despite being found in several studies. Significantly lower proportions were observed, particularly in East Asia (Fig. 5g). In the case of rs12438025 found in COAD and 7 studies, it was located in the STRC gene and showed the highest variant proportions. In particular, it was very high in Africa (Fig. 5h).

### Selection of DEGs and DMRs in three cancers

From three cancers (TCGA-COAD, TCGA-UCEC, and TCGA-STAD), DEGs and DMRs were selected based on whether the patients had variants. The DEGs and DMRs of TCGA-STAD were not calculated because of the insufficient minimum number of samples in the variant group (*n* < 3; Table 1). DEGs and DMRs in the three cancers were selected based on fold changes and *p*-values. The threshold of fold change was |FC| > 0.2 for TCGA-COAD and TCGA-UCEC, and |FC| > 0.3 for TCGA-STAD. Thresholds of p-values were PV < 0.01 for DEGs in TCGA-COAD and TCGA-UCEC, and PV < 10^−10^ in TCGA-STAD. The thresholds of p-values were PV < 10^−12^ for DMRs in all cases (Table 1).

**Fig. 6:**
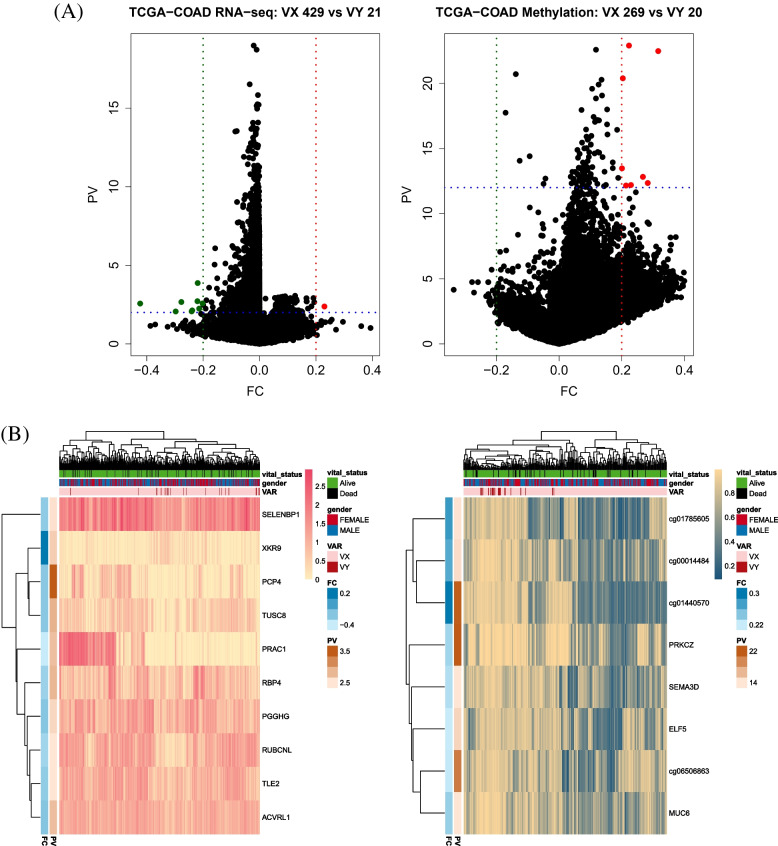
Volcano plots and heatmaps of DEGs (left) and DMRs (right) by variants in TCGA-COAD. The VX means a patient who does not have variants (*n* = 429 in DEGs, *n* = 269 in DMRs) and the VY (*n* = 21 in DEGs, *n* = 20 in DMRs) is the opposite. **a** Volcano plots indicate upregulated (red dots) and downregulated (green dots) DEGs and DMRs in TCGA-COAD RNA-seq data and Illumina 450 k chip analysis in each. Two axes indicate *P*-value and fold change between two groups which has variants.Ten DEGs meet criteria |FC| > 0.2 and *p*-value < 0.01. Eight DMRs also meet criteria |FC| > 0.2 and p-value < 10^−12^. The dashed green and red lines indicate where |FC| > 0.2, and the dashed blue line means the criteria of *p*-values. **b** Heatmaps of DEGs and DMRs representing differences between vital status, gender, and presence of variants. Vital status, gender, and presence of variants are indicated as column annotation bars. Other annotation bars indicate fold change and *p*-value between the two groups

For TCGA-COAD, 10 DEGs were selected (SELENBP1, XKR9, PCP4, TUSC8, PRAC1, RBP4, PGGHG, RUBCNL, TLE2, ACVRL1) and eight DMRs were selected (cg01785505, cg00014484, cg01440570, PRKCZ, SEMA3D, ELF5, cg06506363, MUC6). In the DEG analysis, only one gene was overexpressed in the variant group, and in the DMR analysis, there was no CpG site that was underexpressed in the variant group. The most overexpressed gene in the variant group was XKR9 and the most hypermethylated CpG site was cg01440570 (Fig. 6).

**Fig. 7:**
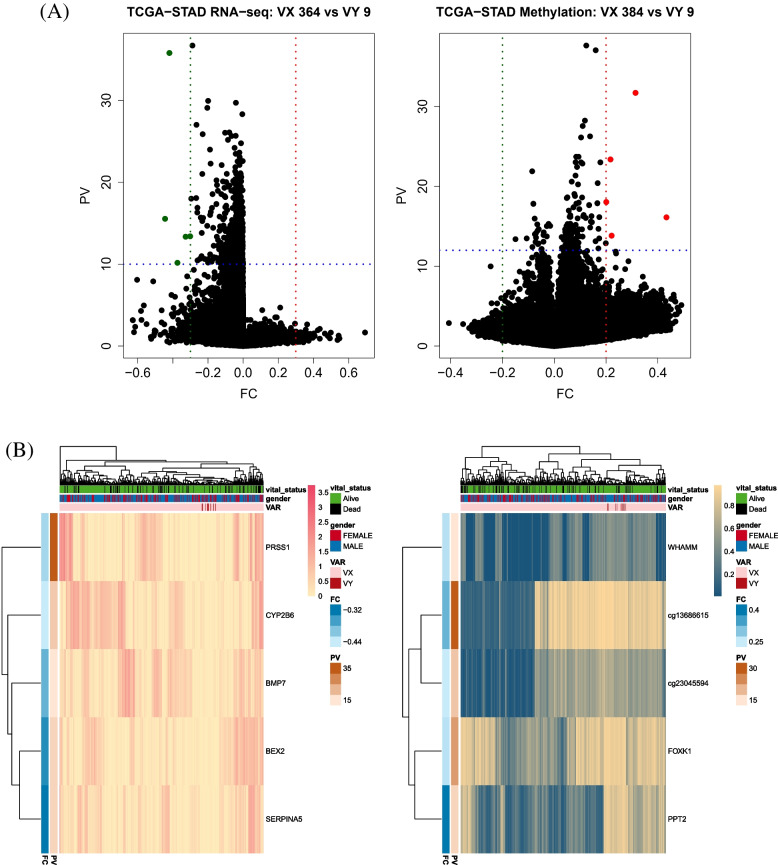
Volcano plots and heatmaps of DEGs (left) and DMRs (right) of patients with variants and without variants in TCGA-STAD. The VX means a patient who does not have variants (*n* = 364 in DEGs, *n* = 384 in DMRs) and the VY (*n* = 9 in DEGs, *n* = 9 in DMRs) is the opposite. In the volcano plot, red dots indicate DEGs and DMRs with increased expression or methylation levels in the variants containing (VY) group. The dashed blue line represents where *P* < 10^−10^ for DEGs and P < 10^−12^ for DMRs. Patients without variants are denoted as VX and patients with variants are denoted as VY. The colored dots were provided as total five DEGs and five DMRs, which were listed as two heatmaps. In heatmap, vital status, gender, and variants are indicated as column annotation bars Two row annotation bars indicate *P*-value and fold change between two groups

For TCGA-STAD, five DEGs (PRSS1, CYP2B6, BMP7, BEX2, and SEPRINA5) and five DMRs (WHAMM, cg13686615, cg23045594, FOXK1, and PPT2) were selected. The most underexpressed gene in the variant group was CYP2B6, and the most hypermethylated CpG site was located in PPT2 (Fig. 7).

**Fig. 8:**
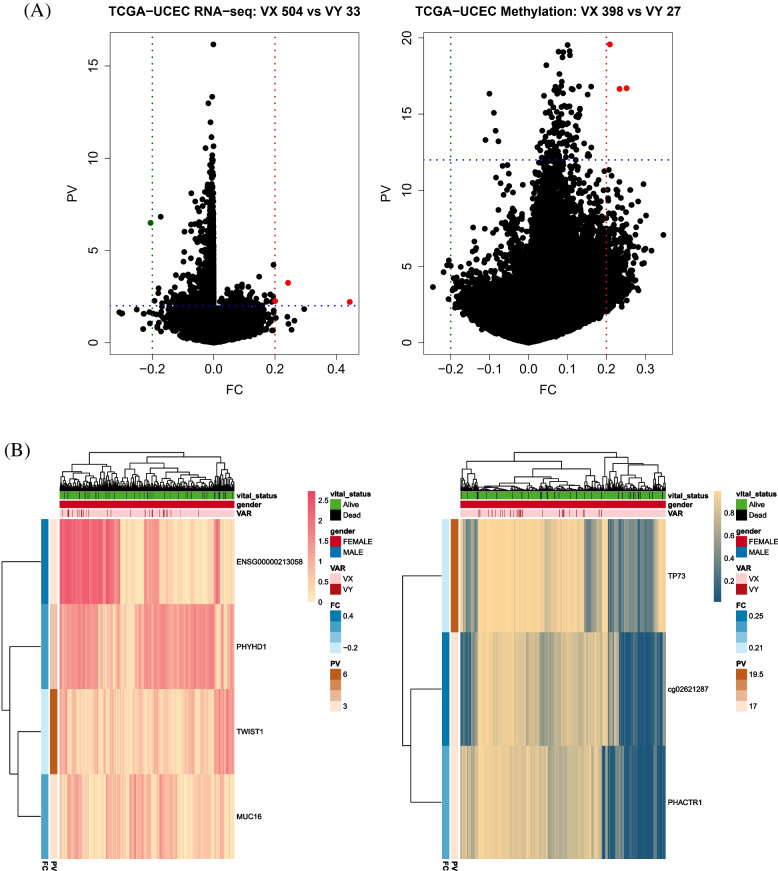
Volcano plots and heatmaps of DEGs (left) and DMRs (right) of patients with variants and without variants in TCGA-UCEC. No variant group (*n* = 504), and variant group (*n* = 33) patients samples were analyzed for DEGs. No variant group (*n* = 398), and variant group (*n* = 27) patients samples were analyzed for DMRs. **a** Four DEGs meet criteria |FC| > 0.2 and p-value < 0.01. Three DMRs also meet criteria |FC| > 0.2 and p-value < 10^−12^. The dashed green and red lines indicate where |FC| > 0.3, and the dashed blue line means the criteria of *p*-values. **b** Heatmaps of DEGs and DMRs representing differences between vital status, gender, and presence of variants

For TCGA-UCEC, four DEGs (ENSG0000213058, PHYHD1, TWIST1, and MUC16) and three DMRs (TP73, cg02621287, and PHACTR1) were selected. The gene with the most statistically significant difference between the two groups was TWIST1 in RNA-seq, and the CpG site was located in the TP73 gene in the methylation analysis (Fig. 8).

### eQTL and mQTL analysis

**Fig. 9:**
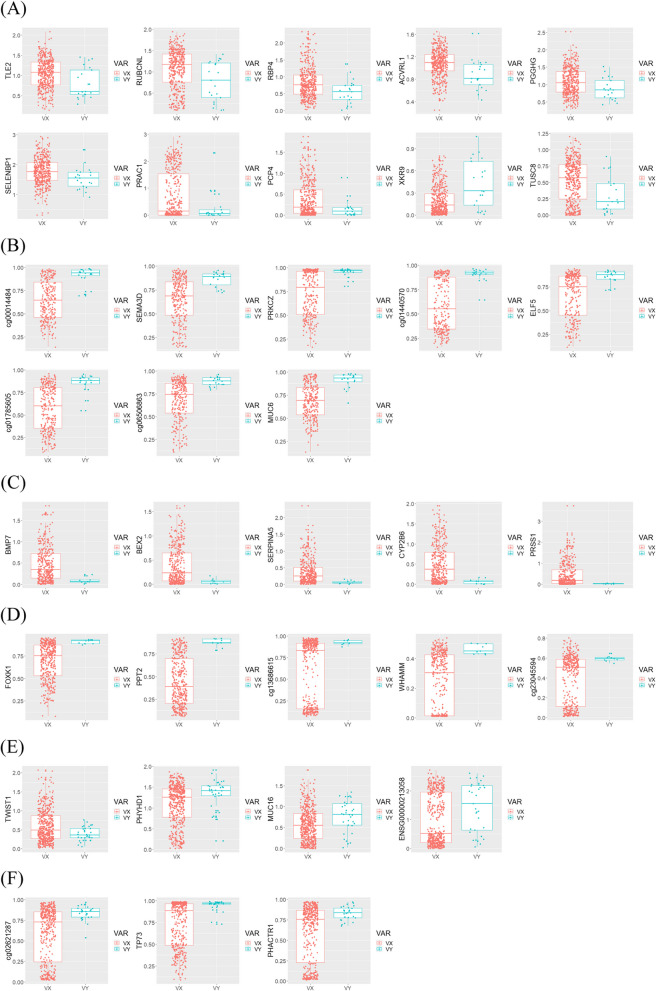
Boxplots of eQTL from RNA-seq data and mQTL from the Illumina 450 k chip. Two groups were divided by variants, and “VX” and “VY” means the absence and presence of variants, respectively. **a** Total 10 genes of eQTL, and (**b**) total eight CpG sites of mQTL results in TCGA-COAD. **c** Total five genes of eQTL, and (**d**) total five CpG sites of mQTL results in TCGA-STAD. **e** Total four genes of eQTL, and (**f**) total three CpG sites of mQTL results in TCGA-UCEC

The eQTL and mQTL analyses were conducted on the genes identified in the DEG and DMR analyses. For a total of three cancers, boxplots are presented for genes that are presented in heatmaps by variants. For TCGA-COAD, 10 DEGs (Fig. 9a; 10 genes) and eight DMRs (Fig. 9 b; eight CpG sites) were analyzed. For TCGA-STAD, five DEGs (Fig. 9 c; five genes) and five DMRs (Fig. 9 d; five CpG sites) were analyzed. Finally, for TCGA-UCEC, four DEGs (Fig. 9 e; four genes) and three DMRs (Fig. 9f; three CpG site) were analyzed. All DEGs were identified from RNA-seq data, and DMRs were obtained from the Illumina 450 k chip. Two groups were separated by the presence or absence of variants (Fig. 9).

### Decision tree for survival prediction

**Fig. 10:**
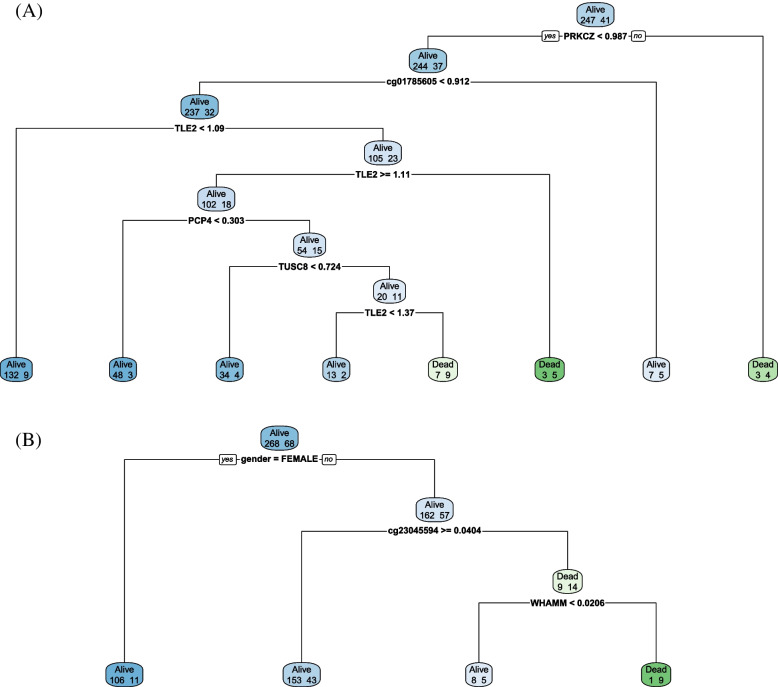
Decision trees for (**a**) TCGA-COAD and (**b**) TCGA-STAD. Decision trees were performed to discover the optimal classification of survival status of tumor patients

A decision tree was designed to determine survival for the three cancer types. The expression or methylation of each gene presented in the heatmap and QTL were targeted as input features. Two clinical features, sex and age, were used as input features. Therefore, two clinical features, along with 18 genomic features in TCGA-COAD, 10 in STAD, and seven features in UCEC, were used to distinguish survival. No decision tree has been designed for TCGA-UCEC. In TCGA-COAD and TCGA-STAD, which are digestive cancers, survival was confirmed with seven and three nodes, respectively (Fig. 10).

## Discussion

High proportions of variants in the cancer genome are derived from somatic variants, whereas most variants from chronic diseases are from germline variants. Therefore, variants related to chronic diseases and cancers are considered to have a low relevance. Nevertheless, the relationship between the variants could be an important factor in the treatment of cancer and chronic diseases.

Cancer and germline variants are related [22], and a variety of evidences have emerged. For example, genes such as BRCA are affected by germline variants. In particular, germline variants in eQTL and mQTL affect cancer progression and patient survival [23]. In addition, GWAS has shown that variants are related to chronic diseases and cancer prognosis [1, 2, 4]. Therefore, in this study, we aimed to identify cancer-related factors from a chronic disease-related variant database (LocusZoom) and TCGA.

This study revealed germline variants in three cancers related to somatic variants from the clinical data of patients with chronic disease using statistical analysis. There were statistically significant variants in the three cancer types. SELENBP1, XKR9, PCP4, TUSC8, PRAC1, RBP4, PGGHG, RUBCNL, TLE2, and ACVRL1 were identified as DEG, and cg01785505, cg00014484, cg01440570, PRKCZ, SEMA3D, ELF5, cg06506363, and MUC6 CpG sites or genes were observed as DMRs of COAD. PRSS1, CYP2B6, BMP7, BEX2, and SEPRINA5 were identified as DEG and WHAMM, cg13686615, cg23045594, FOXK1, and PPT2 CpG sites, and genes were observed in the DMRs of STAD. ENSG0000213058, PHYHD1, TWIST1, and MUC16 genes were identified as DEG and ​​TP73, cg02621287, and PHACTR1 CpG sites or genes were observed in the DMRs of UCEC. In QTL analysis, the expression or methylation levels of each gene are presented as boxplots by variant.

COAD can be classified into four subtypes (CIN, EBV, MSI, and GS), and the different subtype proportions and variant patterns were revealed by region [24]. Therefore, a world map was presented to present the location and proportion of the 11 variants for each population. As shown in the results, the variants showed different rates in each population. Therefore, we can expect ancestral differences to appear in the chronic diseases and cancer characteristics associated with the selected variants. This hypothesis should be further tested with a larger dataset and validated using experimental methods from COAD tissues in different regions. Eight variants were found in the 1000 Genomes Project, of which only two variants were found in STAD and UCEC. The variants were found at a rare rate in a total of 26 populations of the 1000 Genomes Project. This means that compared to STAD and UCEC, mutations related to COAD show relatively greater differences depending on the population.

Decision trees were used to classify the survival status of the patients with cancer. The decision tree results showed that the selected DEGs and DMRs explained the survival prediction. We concluded that chronic disease-related variants were associated with at least two cancers. Therefore, the analysis results and methods of this study can be used for cancer progression research, patient prognosis prediction, and diagnosis [25]. In addition, from the perspective of preventive medicine, this study could help regional cancer and chronic disease prevention, and develop diagnosis strategies.
